# Whole bone marrow cell culture: A convenient protocol for the *in vitro* expansion of endothelial progenitor cells

**DOI:** 10.3892/etm.2014.1827

**Published:** 2014-07-04

**Authors:** JUN-FENG LIU, ZHONG-DONG DU, ZHI CHEN, ZHI-XU HE

**Affiliations:** 1 Laboratory of Tissue Engineering and Stem Cells, Guiyang Medical College, Guiyang, Guizhou 550004, P.R. China; 2 Department of Pediatrics, The General Hospital of Huabei Oil Field Company, Renqiu, Hebei 062552, P.R. China; 3 Department of Cardiology, Beijing Children’s Hospital, Capital Medical University, Beijing 100045, P.R. China

**Keywords:** endothelial progenitor cells, bone marrow, mouse, model

## Abstract

The number and function of endothelial progenitor cells (EPCs) may be a predictive factor for the severity and outcome of cardiovascular disease. However, the manipulation of bone marrow mononuclear cell (BMMC) cultures for EPCs is an elaborate and difficult procedure in small experimental animals. The present study aimed to assess the feasibility of whole bone marrow cell (WBMC) culture for expanding EPCs in small experimental animals. C57BL/6 mice (age, 3–4 weeks; weight, 9.47±0.76 g) were used as the experimental animals, and WBMCs were isolated from the femora and tibiae and cultured in endothelial cell growth medium-2. A BMMC culture for EPCs was used as a control. EPC growth, phenotype and functions were assessed *in vitro* and *in vivo*. The results demonstrated that EPCs were easily obtained from a WBMC culture *in vitro*. The cells exhibited similar growth and biological characteristics when compared with the EPCs derived from the traditional BMMC culture system. Thus, the cells were able to simultaneously bind to lectin and cause phagocytosis of acetylated-low density lipoproteins. In addition, the cells exhibited high expression levels of cluster of differentiation 34 and fetal liver kinase 1, and possessed similar functional properties to BMMC-derived EPCs, including vascular network formation, proliferation, adhesion and migration abilities *in vitro*. Thus, WBMC-derived EPCs can improve the outcome of pulmonary vascular disease when transplanted into a monocrotaline-induced pulmonary hypertension mouse model. The results of the present study indicated that the WBMC culture system is a more convenient and effective method of obtaining and expanding EPCs compared with BMMC culture, with the advantage of a simplified procedure.

## Introduction

The preservation of vascular endothelial integrity is a dynamic process involving the injury and repair of endothelial cells. Vascular endothelial injury and dysfunction is considered to be the first and crucial step in the development of cardiovascular disease (1–3). Restoring the injured endothelium quickly and effectively may prevent or reverse disease progression. Previous studies have indicated that endothelial progenitor cells (EPCs), which are the precursors of endothelial cells, play a pivotal role in vascular homeostasis and endothelial repair, and have been implicated in vasculogenesis or neovascularization associated with cardiovascular disease (4–7). Under conditions of tissue ischemia or injury, EPCs may be mobilized from the bone marrow into the peripheral blood (5), where they migrate to sites of injured endothelium and differentiate into endothelial cells (6). This stimulates angiogenesis and endothelial cell repair (7). A number of studies have indicated that patients with cardiovascular disease, including pulmonary hypertension (PH) and coronary artery disease, exhibit a reduced EPC number and function (8,9). Thus, the upregulation of EPCs may improve the outcome of disease (9), and the EPC number and function may be used as predictive factors for the severity and outcome of cardiovascular disease.

EPCs were first isolated from human peripheral blood using magnetic bead selection (10). Succeeding studies revealed that blood from the umbilical cord or bone marrow contain a more abundant source of EPCs (11,12). To obtain and expand EPCs *in vitro* for functional assessment, mononuclear cells are isolated from the peripheral blood or bone marrow by Ficoll density gradient centrifugation for further culture. However, in order to avoid cell loss and the influence of the procedure on EPC function, the manipulation of this technique is elaborate and difficult in small experimental animals. Thus, a more convenient and effective method may be preferable. A previous study indicated that whole bone marrow cell (WBMC) culture may expand the quantity of mesenchymal stem cells with normal functions (13). Thus, it was hypothesized that a similar technique may also be suitable for EPCs.

To assess the feasibility of WBMC cultures for expanding EPCs in small experimental animals, C57BL/6 mice (age, 3–4 weeks) were used as the experimental animals in the present study. WBMCs were isolated from the femora and tibiae and cultured in endothelial cell growth medium-2 (EGM-2; Lonza Systems, Basel, Switzerland). The growth, phenotype and function of the EPCs were assessed *in vitro* and *in vivo*.

## Materials and methods

### Animals

C57BL/6 male mice, either aged 3–4 weeks with a body weight of 9.47±0.76 g or aged 6–8 weeks with a body weight of 23.35±2.74 g, were purchased from the Capital Medical University (SCXK2005-0006; Beijing, China) and housed under specific pathogen-free conditions in the Department of Laboratory Animals at the Capital Medical University. All animal studies and procedures were approved by the Institutional Animal Care and Use Committee of the Capital Medical University.

### WBMC culture for EPCs

A total of 12 mice (age, 3–4 weeks) were randomly divided into two groups (n=6 per group) for the WBMC and bone marrow mononuclear cell (BMMC) cultures. The mice were sacrificed by decapitation and the femora and tibiae of the mice were harvested aseptically. WBMCs were flushed using phosphate-buffered saline (PBS) in a 1-ml syringe with a 25-gauge needle. Following collection of the cells by centrifugation at 300 × g for 5 min, the WBMCs were resuspended at a density of 10^7^ cells/ml in EGM-2. The medium included endothelial cell basal medium-2 (EBM-2), supplemented with 5% fetal bovine serum (FBS), basic fibroblast growth factor, vascular endothelial growth factor, epidermal growth factor, recombinant insulin-like growth factor, ascorbic acid and gentamicin/amphotericin-B. The cell suspension was plated into 24-well culture plates (1 ml/well), pre-coated with fibronectin (Sigma-Aldrich, St. Louis, MO, USA), and incubated at 37°C in a humidified environment with 5% carbon dioxide (CO_2_). Half of the medium was renewed every day for the first three days, and then replaced every two days thereafter.

Following seven days of culture, the cells were incubated for 4 h with 10 μg/ml 1,10-dioctadecyl-3,3,30,30-tetramethylindocarbocyanine perchlorate-labeled acetylated low-density lipoprotein (DiI-acLDL; Molecular Probes^®^; Invitrogen Life Technologies, Carlsbad, CA, USA). Next, the cells were fixed with 4% paraformaldehyde and incubated for 1 h with 10 μg/ml fluorescein isothiocyanate (FITC)-conjugated lectin (Sigma-Aldrich). After washing extensively with PBS, the cells were examined by confocal laser scanning microscopy to identify the EPCs, as described previously (12). Furthermore, the cell-surface markers, cluster of differentiation (CD)34 and fetal liver kinase 1 (Flk-1), were analyzed by flow cytometry (BD Biosciences, Franklin Lanes, NJ, USA). Briefly, the cells were detached using 0.25% trypsin and 0.04% ethylenediaminetetraacetate acid (EDTA), and resuspended in 200 μl PBS. The cells were incubated for 20 min in the dark at 4°C with 2 μl FITC-conjugated rat monoclonal antibody against mouse Flk-1 (BD Biosciences) and 5 μl phycoerythrin (PE)-conjugated rat monoclonal antibody against mouse CD34 (BD Biosciences). The samples were centrifuged at room temperature for 5 min at 300 × g, resuspended in 500 μl PBS and evaluated using flow cytometry. Furthermore, a BMMC culture for the EPCs was performed as described previously (12), to be used as the control.

### RNA isolation, reverse transcription and quantitative polymerase chain reaction (qPCR)

Total RNA of the EPCs was extracted using an E.Z.N.A.^®^ Total RNA kit I (Omega Bio-Tek, Norcross, GA, USA) and reverse transcribed to cDNA using an M-MLV Reverse Transcriptase kit (Invitrogen Life Technologies). The qPCR analysis for endothelial nitric oxide synthase (eNOS) was performed with Platinum^®^ SYBR^®^ Green qPCR SuperMix-UDG w/ROX (Invitrogen Life Technologies) with an Applied Biosystems^®^ 7300 Real-Time PCR system (Invitrogen Life Technologies). The PCR primer sequences were as follows: GAPDH forward, 5′-AGCCTCGTCCCGTAGACAAAA-3′ and reverse, 5′-TGGCAACAATCTCCACTTTGC-3′; eNOS forward, 5′-TGTCACTATGGCAACCAGCGT-3′ and reverse, 5′-GCGCAATGTGAGTCCGAAAA-3′.

### EPC functions in vitro

To assess the functions of EPCs *in vitro*, EPCs obtained from the bone marrow following seven days of culture were separated using 0.25% trypsin and 0.04% EDTA. Functions, including vascular network formation, proliferation, adhesion and migration, were assessed as described previously (12,14). With regard to the vascular network formation ability *in vitro*, the EPCs were suspended in EGM-2 at a density of 10^5^ cells/ml (100 μl/well), cultured in 96-well round-bottomed plates that had been pre-coated with Matrigel^™^ (BD Biosciences) and incubated at 37°C in a humidified environment with 5% CO_2_. Images were captured using a light microscope (14). To determine the proliferation ability, 200 μl EPC suspension at a density of 10^5^ cells/ml in EGM-2 was cultured in 96-well round-bottomed plates for 24 h. Subsequently, 20 μl thiazolyl blue (5 g/l) was supplemented for the 4-h culture. At the end of culture, the supernatant was discarded and 100 μl dimethyl sulfoxide was added for a 10-min incubation. Absorbance was measured at a wavelength of 490 nm. To measure the adhesion ability, 500 μl EPC suspension, at a density of 10^5^ cells/ml in EGM-2, was plated in 24-well plates that had been pre-coated with fibronectin and cultured for 30 min. Following washing three times with PBS, the attached EPCs were counted. To determine the migration ability, 100 μl EPC suspension, at a density of 5×10^5^ cells/ml in EBM-2 + 0.5% FBS, were plated in the upper chamber of a modified Boyden chamber (8 μm pore size; Corning, Tewksbury, MA, USA), and placed in 24-well plates containing 600 μl EGM-2/well. Following incubation for 24 h, the cells on the lower membrane were fixed with 4% paraformaldehyde and stained with 0.1% crystal violet. The migrated EPCs were counted.

### EPC function in vivo

To identify the functions of the EPCs derived from the WBMC culture, a mouse model of PH was established to assess the therapeutic effects of EPC transplantation. A total of 24 mice (age, 6–8 weeks) were randomly divided into four groups (n=6 per group): PH model, WBMCs-EPCs transplantation, BMMCs-EPCs transplantation and the control. PH was induced by a single subcutaneous injection of monocrotaline (MCT; 60 mg/kg; Sigma-Aldrich) (9), while PBS was administered to the mice in the control group. At day five following the injection of MCT, the EPCs were transplanted into the mice. For transplantation, the EPCs were detached using 0.25% trypsin and 0.04% EDTA, and resuspended in 1 ml PBS at a density of 10^6^ cells/ml. The cells were subsequently transplanted into the mice via the caudal vein. To further observe the distribution of transplanted EPCs in the lungs, two additional mice received transplantion of EPCs pre-labeled with CellTracker^™^ CM-DiI (Invitrogen Life Technologies) using the same procedure. At day 16 following the transplantation of EPCs, the mice were sacrificed by decapitation and the lung tissue was removed. The tissue was fixed in 10% paraformaldehyde for 24 h at room temperature and embedded in paraffin. Serial 5-μm sections were stained with hematoxylin and eosin, and observed under a microscope. The medial wall thickness (WT) of the pulmonary arterioles was assessed according to a method described in a previous study (9).

### Statistical analysis

Data are presented as the mean ± standard deviation. SPSS 17.0 software (SPSS, Inc., Chicago, IL, USA) was used to conduct the statistical analyses. Differences were compared using the independent t-test or one-way analysis of variance, where P<0.05 was considered to indicate a statistically significant difference.

## Results

### Growth of EPCs in vitro

**Figure 1:**
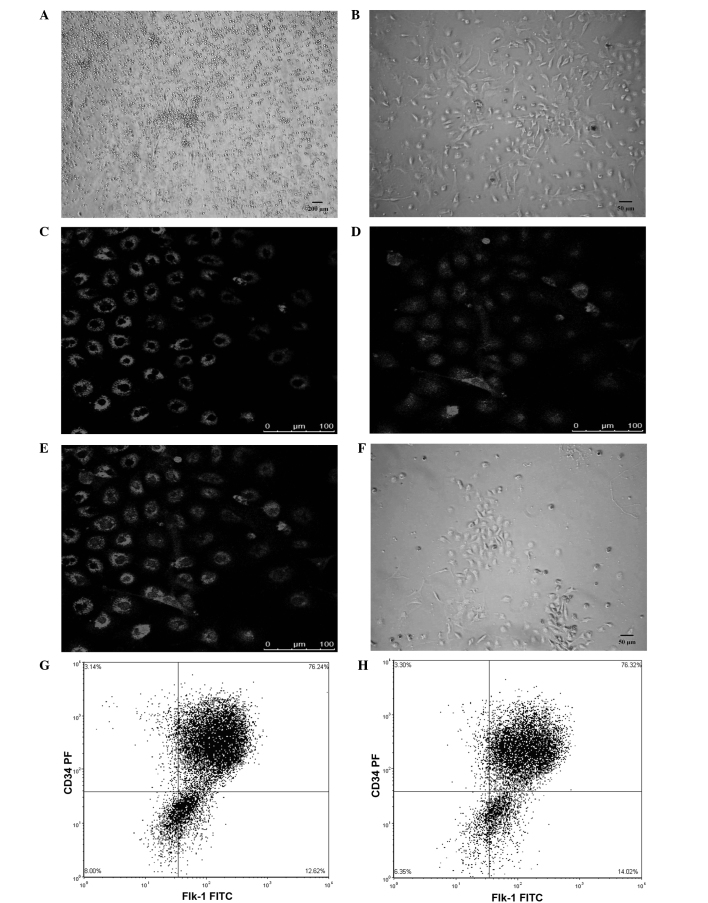

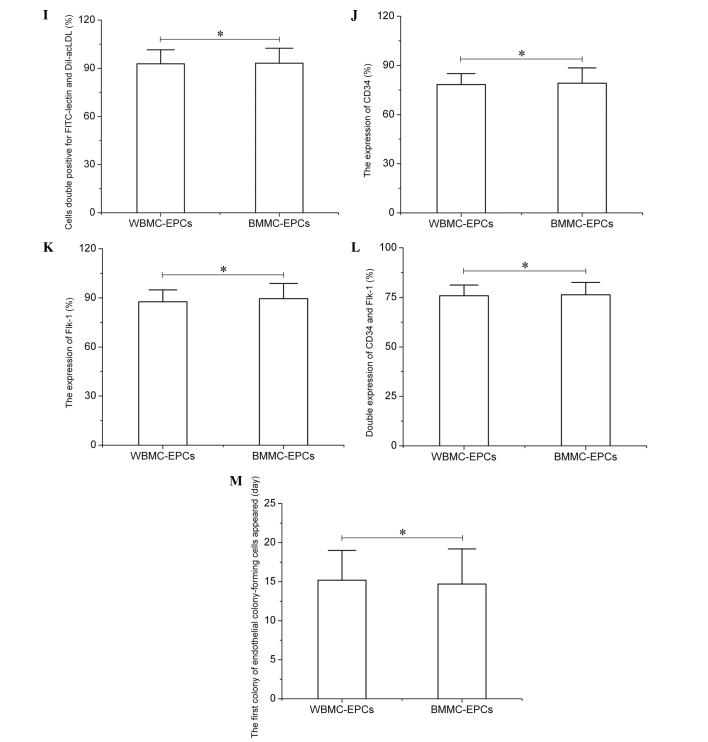
Whole bone marrow cell (WBMC) culture for endothelial progenitor cells (EPCs) and their identification *in vitro*. (A) Following two days of culture, a small ‘blood island’ surrounded by spindle-like cells was observed. (B) Following seven days of culture, cells exhibited a spindle-like appearance. The majority of the cells were immunopositive for (C) 1,10-dioctadecyl-3,3,30,30-tetramethylindocarbocyanine perchlorate-labeled acetylated low-density lipoprotein (DiI-acLDL) and (D) FITC-conjugated Flk-1. (E) Cells positive for FITC-lectin and DiI-acLDL were recognized as EPCs undergoing differentiation. (F) Following cell culture for ~2 weeks, endothelial colony-forming cells, which originated from the EPCs, appeared and exhibited a typical cobblestone morphology. Cells expressed CD34 and Flk-1 in the (G) WBMC and (H) bone marrow mononuclear cell culture systems. FITC, fluorescein isothiocyanate; Flk-1, fetal liver kinase 1; CD, cluster of differentiation. WBMC culture for EPCs and their identification *in vitro*. Comparison between WMBC-EPCs and BMMC-EPCs with regard to the expression levels of (I) double fluorescence positive cells for DiI-acLDL and FITC-Flk-1, (J) CD34, (K) Flk-1 and (L) double fluorescence positive cells for CD34 and Flk-1, and (M) the time that the first endothelial colony-forming cells appeared. Data (n=6) are presented as the mean ± standard deviation.^*^P>0.05. WBMC, whole bone marrow cell; BBMC, bone marrow mononuclear cell; EPCs, endothelial progenitor cells; DiI-acLDL, 1,10-dioctadecyl-3,3,30,30-tetramethylindocarbocyanine perchlorate-labeled acetylated low-density lipoprotein; FITC, fluorescein isothiocyanate; Flk-1, fetal liver kinase 1; CD, cluster of differentiation.

Following two days of culture, a small ‘blood island’ surrounded by spindle-like cells was observed in the WBMC culture system (Fig. 1A). After seven days of culture, the cells adhered to the base of the culture plate and exhibited a spindle-like appearance (Fig. 1B). Using immunofluorescence labeling with FITC-lectin and DiI-acLDL, the majority of cells were shown to be positive for FITC-lectin and DiI-acLDL using confocal laser scanning microscopy (Fig. 1C–E); thus, were defined as EPCs undergoing differentiation (12). There was no difference in the number of double fluorescence positive cells between the WBMC and BMMC culture systems (92.8±8.7 vs. 93.2±9.3%; P>0.05; Fig. 1I). Furthermore, the expression levels of CD34 and Flk-1, as analyzed by flow cytometry, revealed no statistically significant differences between the WBMC and BMMC culture systems (CD34, 78.3±6.7 vs. 79.2±8.6%; Flk-1, 87.6±7.3 vs. 89.5±8.5%; double expression of CD34 and Flk-1, 75.8±5.4 vs. 76.3±6.2%; P>0.05; Fig. 1G, H and J–L). In the subsequent culture of approximately one week, endothelial colony-forming cells, which originated from the EPCs (15), gradually appeared (Fig. 1F). These cells exhibited a typical cobblestone morphology and proliferated rapidly. The first colony was detected following 15.2±3.8 days of culture in the WBMC culture system and no statistically significant differences were identified when compared with the BMMC culture system (14.7±4.5 days; P>0.05; Fig. 1M).

### Expression levels of eNOS

**Figure 2:**
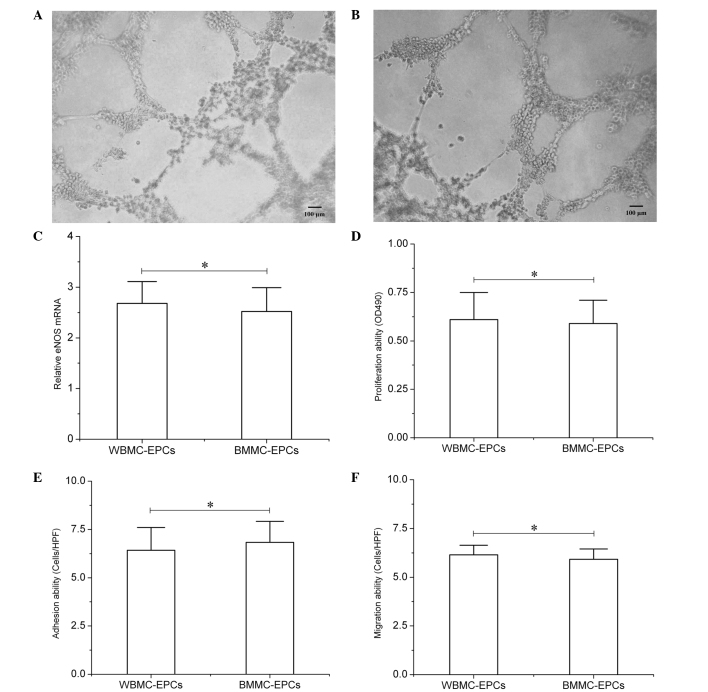
EPC functions *in vitro*. EPCs derived from (A) BMMC or (B) WBMC culture systems demonstrated a marked morphological change with the cells connecting to each other to form two-dimensional networks following 5 h of culture in 96-well round-bottomed plates that were pre-coated with Matrigel. (C) Expression levels of eNOS, and (D) proliferation, (E) adhension and (F) migration abilities of the EPCs. Data (n=6) are presented as the mean ± standard deviation.^*^P>0.05. WBMC, whole bone marrow cell; BBMC, bone marrow mononuclear cell; EPCs, endothelial progenitor cells; eNOS, endothelial nitric oxide synthase; OD, optical density; HPF, high power field.

qPCR analysis demonstrated that the EPCs derived from the WBMC culture system expressed similar levels of eNOS to those from the BMMC culture system (^ΔΔ^Ct 2.68±0.43 vs. 2.52±0.47; P>0.05; Fig. 2C).

### EPC functions in vitro

To assess the vascular network formation ability, the EPCs derived from the BMMC culture system were plated in 96-well round-bottomed plates that had been pre-coated with Matrigel. Following ~5 h of culture, the EPCs revealed a marked morphological change, with the cells connecting to each other to form two-dimensional networks (Fig. 2A). The same vascular network formation was also observed in the EPCs derived from the WBMC culture system (Fig. 2B). Furthermore, analyses of EPC proliferation, adhesion and migration ability *in vitro* were performed to assess the functional status of the EPCs. The results did not reveal any statistically significant differences between the two culture systems [WBMC culture system: Proliferation optical density (OD)_490_, 0.61±0.14; adhesion, 6.42±1.18 cells/high power field (HPF); migration, 6.15±0.49 cells/HPF; BMMC culture system: Proliferation OD_490_, 0.59±0.12; adhesion, 6.83±1.09 cells/HPF; migration, 5.92±0.53 cells/HPF; P>0.05; Fig. 2D–F).

### EPC functions in vivo

**Figure 3:**
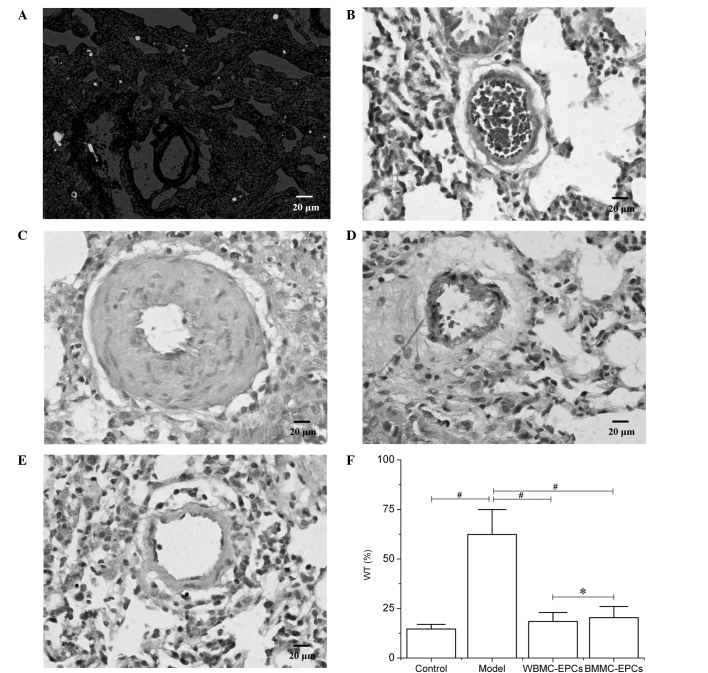
EPC functions *in vivo*. (A) Two days following the transplantation of CM-DiI-labeled EPCs, an abundance of CM-DiI positive cells were observed in the lungs. (B–E) Optical photomicrographs of the lungs stained with hematoxylin and eosin in the (B) control, (C) model, (D) WBMC-EPCs transplant and (E) BMMC-EPCs transplant groups. (F) Changes in the WT in each group. Data (n=6) are presented as the mean ± standard deviation. ^#^P<0.01 and ^*^P>0.05. WBMC, whole bone marrow cell; BBMC, bone marrow mononuclear cell; EPCs, endothelial progenitor cells; WT, wall thickness.

A mouse model of PH was used to further assess the therapeutic effects of EPCs *in vivo*. The results revealed that two days following the transplantation of CM-DiI-labeled EPCs, an abundance of cells positive for CM-DiI were observed in the lungs (Fig. 3A). At day 21 following the subcutaneous injection of MCT, histological examination of the lungs indicated that medial hypertrophy of the pulmonary muscular arterioles was evident in the PH model group (Fig. 3C). In addition, the WT increased in the model group when compared with the control group (62.37±12.58 vs. 14.62±2.35%; P<0.01; Fig. 3F). However, transplantation of EPCs derived from the BMMC and WBMC culture systems improved the medial hypertrophy of the pulmonary muscular arterioles (Fig. 3D and E). The WT in the two transplantation groups decreased when compared with the model group and no statistically significant differences were observed between the two transplantation groups (18.46±4.52 vs. 20.37±5.63%; P>0.05; Fig. 3F).

## Discussion

The present study demonstrated that EPCs were easily obtained from WBMCs cultured *in vitro*. The cells exhibited similar growth and biological characteristics to EPCs derived from a traditional BMMC culture system. Thus, the EPCs were able to simultaneously bind to lectin and cause phagocytosis of acLDLs. In addition, the cells exhibited high expression levels of CD34 and Flk-1, possessed similar functional properties to BMMC-derived EPCs *in vitro* and improved the outcome of pulmonary vascular disease when transplanted into a mouse model. These characteristics indicate that the WBMC culture system is a more convenient and effective method of obtaining EPCs, with the advantage of a simplified procedure. Thus, this strategy may allow an improved understanding of the EPC status in a number of vascular diseases, particularly in small experimental animal models, and an improved evaluation of the severity and outcome of the disease.

Numerous studies have indicated that changes to the EPC number and function participate in the pathogenesis of cardiovascular disease. Analysis of the EPC number and function during the early stages of disease may improve the evaluation of disease severity and outcome (8,9). The outcome of the disease may be improved via the upregulation of EPCs (9,16,17). Thus, studies investigating EPCs in cardiovascular disease have attracted increasing attention. Directly isolating a sufficient number of EPCs from the peripheral blood for functional analysis is difficult due to the low abundance of EPCs. Thus, EPC expansion in a specific culture medium is commonly required. Since the first isolation of EPCs from the peripheral blood (10), EPCs have subsequently been obtained from umbilical cord blood, bone marrow, liver, tunica externa and adipose tissue (11,12,18–21). Among the diverse sources of EPCs, peripheral blood remains the most common option for further mononuclear cell isolation and culture. However, this procedure is not easily performed in small experimental animals, as the EPC content of the peripheral blood is low. Thus, bone marrow, which is a reservoir of circulating EPCs, is normally used as a substitute. However, the procedure remains difficult in small experimental animals at a very young age since the EPC content in their bone marrow cells is too low for adequate collection. Furthermore, the procedure of density gradient centrifugation further aggravates cell loss. Thus, a more convenient and effective method is required.

The procedure of WBMC culture for mesenchymal stem cells (13) indicates that a similar technique may also be suitable for EPCs. Based on this hypothesis, in the present study, WBMCs segregated from mice aged 3–4 weeks-old and with a low body weight, were cultured in endothelial cell growth medium to observe the growth of EPCs. The results revealed that following seven days of culture, there was an abundance of EPCs with a spindle-like appearance. The majority of these EPCs were undergoing differentiation, with the characteristics of simultaneous lectin binding and acLDL phagocytosis, as well as high expression levels of specific membrane molecules, including CD34 and Flk-1. Compared with the traditional BMMC culture procedure, there were no differences with regard to EPC characteristics, indicating the feasibility of WBMC culture for expanding EPCs in small experimental animals. Furthermore, in the subsequent culture period, endothelial colony-forming cells gradually appeared and there was no difference in the time point of the first colony appearance between the two culture systems. Endothelial colony-forming cells are considered to be important descendants of EPCs that play a crucial role in the repair process of injured vascular endothelium (15). The similarity in growth characteristics indicates that the EPCs derived from the WBMC culture system may play a similar function in vascular endothelial repair to those from the BMMC culture system. To further confirm this hypothesis, EPC functions *in vitro*, including vascular network formation, proliferation, adhesion and migration, were assessed. The results did not reveal any differences between the two culture systems. Furthermore, previous studies have indicated that nitric oxide, an important regulatory molecule primarily produced by EPCs or endothelial cells, may play a pivotal role in cardioprotective effects (22,23). Thus, the normal expression level of eNOS is a crucial index for the EPC functional status. The present study demonstrated that there was no statistically significant difference in the expression levels of eNOS between EPCs derived from the WBMC and the traditional BMMC culture systems. Therefore, the WBMC culture system is a convenient and reliable procedure for the *in vitro* study of EPCs.

To further analyze the *in vivo* function of EPCs derived from the WBMC culture system, a mouse model of PH was established to assess the therapeutic effect of EPC transplantation. Previous studies have confirmed that EPC transplantation is effective in preventing the progression of PH in laboratory animal models (24,25). Thus, the use of a PH model in the current study was a reliable method for assessing EPC function *in vivo*. The results revealed that the transplanted EPCs were able to successfully migrate to the lungs, improving the medial hypertrophy of the pulmonary muscular arterioles in the MCT-induced mouse model of PH. There were no differences in the WT between the transplantation of EPCs derived from the WBMC and the traditional BMMC culture systems, indicating that the EPCs obtained by the WBMC culture procedure possessed the same functional characteristics as the EPCs obtained by the BMMC culture system. Thus, the WBMC culture system is suitable for assessing the *in vivo* function of EPCs.

In conclusion, the results of the present study demonstrated that the WBMC culture system is a convenient and effective method of obtaining and expanding EPCs, with the advantage of a simplified procedure and normal function. However, the reliability of this technique remains controversial and requires further investigation for the future treatment of cardiovascular disease, particularly in small experimental animal models.
